# Sex dependent effects of post-natal penicillin on brain, behavior and immune regulation are prevented by concurrent probiotic treatment

**DOI:** 10.1038/s41598-020-67271-4

**Published:** 2020-06-25

**Authors:** Marya Kayyal, Tanvi Javkar, M. Firoz Mian, Dana Binyamin, Omry Koren, Karen-Anne McVey Neufeld, Paul Forsythe

**Affiliations:** 1 McMaster Brain-Body Institute at St Joseph’s Healthcare Hamilton, Hamilton, Canada; 2 The Azrieli Faculty of Medicine, Bar-Ilan University, Safed, Israel; 3 Department of Pathology and Molecular Medicine, McMaster University, Hamilton, Canada; 4 Department of Medicine, McMaster University, Hamilton, Canada; 5 Firestone Institute for Respiratory Health, St Joseph’s Healthcare Hamilton, Hamilton, Canada

**Keywords:** Immunology, Neuroscience

## Abstract

There is increasing awareness of the need to consider potential long-term effects of antibiotics on the health of children. In addition to being associated with immune and metabolic diseases, there is evidence that early-life antibiotic exposure can affect neurodevelopment. Here we investigated the effect of low dose of penicillin V on mice when administered for 1 week immediately prior to weaning. We demonstrated that exposure to the antibiotic during the pre-weaning period led to long-term changes in social behaviour, but not anxiety-like traits, in male mice only. The change in behaviour of males was associated with decreased hippocampal expression of AVPR1A and AVPR1B while expression of both receptors was increased in females. Spleens of male mice also showed an increase in the proportion of activated dendritic cells and a corresponding decrease in regulatory T cells with penicillin exposure. All changes in brain, behaviour and immune cell populations, associated with penicillin exposure, were absent in mice that received *L. rhamnosus* JB-1 supplementation concurrent with the antibiotic. Our study indicates that post-natal exposure to a clinically relevant dose of antibiotic has long-term, sex dependent effects on the CNS and may have implications for the development of neuropsychiatric disorders. Importantly, we also provide further evidence that probiotic based strategies may be of use in counteracting detrimental effects of early-life antibiotics on neurodevelopment.

## Introduction

Oral antibiotics, specifically the β-lactam group of antibiotics, are the most widely used form of pediatric medication^1^. Antibiotics are administered to over 10% of European children annually^2^ and account for 25% of all the prescriptions written to the pediatric population in the USA.

The development of antibiotics was transformative to medicine, rendering previously lethal infections relatively harmless, and saving millions of lives^3^. However, evidence is emerging that exposure to antibiotics, particularly in early life, may have some detrimental effects on long-term health. Antibiotic administration in childhood has been associated with development of inflammatory bowel disease (IBD)^4^, asthma^5^, obesity^6^ and diabetes^7,8^ later in life. Antibiotics reduce microbial diversity^9,10^ and, given the important role of the microbiota in the maturation and function of many physiological processes, it is suggested that the detrimental effects of early-life administration of antibiotics may be mediated by causing dysbiosis at a critical time point in development.

There is now good evidence, in humans and rodents, for the role of specific microbial compositions in modulating brain function and behaviour^11,12^. Gut microbial maturation and neurodevelopment share critical growth periods^13^ and animal models indicate that early-life events which perturb microbial colonization also influence behaviour in adulthood^14,15^. A better understanding of the impact of early-life antibiotics on the central nervous system (CNS) is required to drive development of strategies to mitigate the potential detrimental effects.

Several studies have shown that cocktails of antibiotics given at high doses, in adult or adolescent mice, induced changes in gut microbiota with associated behavioural alterations^16,17^. However, these combinations of antibiotic are generally used in an attempt to mimic the germ-free state, not to model antibiotic use in clinical practice. It has previously been demonstrated that a low dose of a single antibiotic, penicillin, given to dams during the entire perinatal period (from 1 week before birth to weaning), results in lasting changes in gut microbiota, modified blood-brain barrier integrity in the hippocampus, and reduced anxiety-like and social behaviour in offspring^18^. However, it is not known if there are long-term effects on brain and behaviour from direct exposure to clinically relevant doses of antibiotics for shorter durations, exclusively in the post-natal period. The early postnatal period is associated with growth, as most organs and tissue structures develop prenatally in mammals. However, the CNS is an exception because a considerable amount of morphological maturation occurs postnatally^19^ and postnatal environmental insults may shape this process to determine long-term psychiatric disorders^13^. Given that prenatal penicillin was previously demonstrated to modulate social interactions^18^, we examined whether the postnatal treatment would influence the arginine vasopressin and oxytocin systems that are known to be involved in social behaviour. Specifically, we measured the mRNA expression of several neuropeptide receptors involved in this system. We also examine expression of the neurotrophin, Brain Derived Neurotropic Factor (BDNF), as long-term changes in this neurotrophin have been associated with childhood adversity and linked to emotional regulation, depression, and anxiety-like behaviours^20^. Based on previous studies indicating blood-brain barrier integrity is altered with disruption of the gut microbiota in early life^18,21^, we also assessed expression of mRNA for critical tight junction proteins. As mood disorders and neurodevelopmental disorders, such as autism, occur at different rates between sexes^22^ we investigated effects of postnatal penicillin exposure in both male and female mice. *Lactobacillus rhamnosus* JB-1 (JB-1) has been demonstrated to have psychoactive and neuroactive properties^23,24^ and to attenuate some effects of both stress and antibiotic exposure^18^. We also tested whether concurrent supplementation with JB-1 may counteract the biological and behavioural changes induced by post-natal treatment with low-dose penicillin.

## Methods

### Treatment protocol

Male and female BALB/c mice (breeding pairs), 6–8 weeks old, were acquired from Charles River (Montreal, QC, Canada) and allowed to acclimatize in the housing facility for at least 1 week. For breeding a single female mouse was placed in the male’s cage for 48 h. Pregnancy was confirmed by increased weight (2 g within 8 days following mating). One week before delivery, pregnant females were housed singly with nesting material.

**Figure 1:**
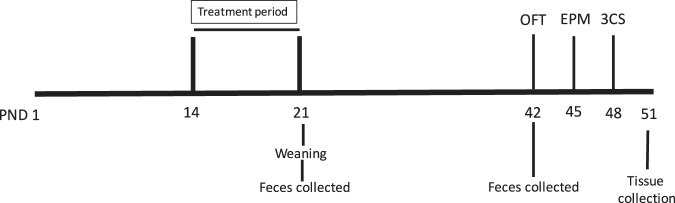
Experimental protocol. PND, post-natal day; OFT, open field test; EPM, elevated plus maze; 3CS, 3 chamber sociability.

Once pups were 14 days old, each cage was randomly assigned to one of three treatment groups: AB, AB + JB1 and control. No more than 3 animals from a litter were assigned to the same group. Pups in the AB group (14 male, 15 female) were treated with PBS in the morning (10 am) and penicillin V (33 mg/kg in PBS) in the afternoon (3 pm). The AB + JB1 group (15 male, 15 female) were fed *L. rhamnosus* JB1 (1 × 10^9^ CFU in PBS) in the morning and penicillin V (33 mg/kg) in the afternoon. The control group (15 male, 15 female) was fed PBS in the morning and the afternoon. Treatments were delivered orally via a 20- gauge plastic feeding tube for 7 consecutive days. The total treatment volume did not exceed 80 μl per day, complying with pup oral feeding guidelines^25^. On the day following the final treatments mice were weaned with male and female offspring separated from dams and housed 3–5 per cage. Weaned mice received drinking water and standard rodent chow *ad libitum*. A battery of behavioural tests was started when the offspring reached 6-weeks old (postnatal day 42), with 48 hours of rest between each test. Fecal samples were collected on the final day of treatment (3 weeks old) and immediately prior to the first behavioral test (6 weeks old). 48 hours following behavioral testing mice were euthanized via decapitation and serum, spleens and brain tissue collected. The treatment protocol is outlined in Fig. 1.

All experiments followed the guidelines of the Canadian Council on Animal Care and were approved by the McMaster Animal Research Ethics Board.

### Behavioral Tests

#### Open field

Mice were tested during the dark phase under dim-light conditions. After a 1 h period of habituation in the testing room, mice were placed singly into an 18 × 38 cm clear Plexiglas enclosure for a period of 30 min. Distance travelled in the open field was used to assess locomotor activity and time spent in the centre of the field was used to assess anxiety-like behaviour. Animal movements were recorded using Motor Monitor software (Kinder Scientific, Poway, CA). The apparatus was cleaned with water and dried between each animal.

#### Elevated plus maze

This test was conducted as we have described previously^18^. Mice tested during the dark phase under dim light conditions. Mice were tested in the EPM apparatus that is elevated at 76 cm off the ground, consists of four arms (length 30 cm, Width 5 cm) —two open arms and two closed arms made with black Plexiglas walls (16 cm). Following habituation period to the testing room for 30 min, the mouse was placed at the intersection of the four arms, facing an open arm, and allowed to explore for 5 min. Behavioural data were analysed using Motor Monitor software. Time spent and number of entries into the open arms were used to assess anxiety-like behaviour. The apparatus was cleaned with water and dried between each animal.

#### Three-chamber sociability test

Mice were tested in the three-chamber apparatus during the light phase, and allowed to habituate to the testing room for 30 min^18^. The apparatus consists of three rectangular Plexiglas chambers, each measuring 24.5 cm L × 44 cm W × 30 cm H, with small openings in the dividing walls that allow access into each chamber. To begin testing the experimental mouse was placed in the centre chamber with the doorways closed and allowed to explore, and habituate to, the chamber for a 5 min period. An unfamiliar mouse (strain- and sex-matched) was then placed within an inverted wire cup in one of the outer chambers while an empty wire cup was placed in the other outer chamber. The doors to the outer chambers were opened for 10 min during which time the experimental mouse was allowed to explore the three chambers. Time spent in each chamber was recorded by a video camera positioned over the apparatus and analysed by using EthoVision XT (Noldus, Leesburg, VA) software. Social interaction ratio was calculated as time spent in social chamber/ time spent in non-social chamber. Social preference is defined as a ratio greater than 0.5.

### Flow cytometry

48 hours following the final behavioral test, spleens were harvested and a single cells suspension of splenocytes prepared for flow cytometry analysis as we have described previously^23,26^. Splenocytes (10^6^) were stained for markers of dendritic cell (DC) maturation and function—CD11c- PerCP-Cy5, MHCII-FITC, CD80-PE, and CD86-APC—or regulatory T cells—CD3-APC, CD4-FITC, CD25-PE-Cy7, and intracellular IL-10-PE (BD Pharmingen, San Diego, CA, USA; eBioscience, San Diego, CA, USA). Following surface staining, cells were fixed and permeabilized with BD Cytofix/Cytoperm before staining for intracellular markers. Data were acquired with FACSCanto (Becton Dickinson, Oakville, ON, Canada) and analysed using FlowJo (TreeStar, Ashland, OR, USA) with doublets and cell debris excluded by FSC and SSC gating. Gating strategies are shown in Fig. S1.

### Cytokine/chemokine analysis

Trunk blood was collected from mice and allowed to clot for 30 min at room temperature. After clotting, the blood was centrifuged at 1000 x g for 10 min at 4 °C. Serum aliquots (25 μl) were placed into a pyrogen/endotoxin-free polypropylene tubes (Thermofisher Scientific, USA) and diluted 2-fold with PBS (25 μl). Samples were stored at −80 °C prior to shipment, on dry ice, to Eve Technologies (Calgary, AB), and analysis conducted for interleukin (IL) -1a, -1b, -2, -4, -5, -6, -10, -12, -13, -17A, chemokine (C-X-C motif) ligand-1, −2, −5, C-C motif ligand-2 (CCL-2), tumor necrosis factor-alpha (TNF-α), and interferon-gamma (IFN-γ) using a multiplex cytokine array. The assay is based on the Luminex technology and utilizes fluorescent color-coded beads pre-coated with capture antibodies targeting 16 specific cytokines^27^. Plasma samples were incubated with the beads prior to addition of biotinylated detection antibodies followed by phycoerythrin (PE)-conjugated streptavidin. Bound cytokines were quantitated using the Bio-Rad BioPlex 200 bead analyzer. This dual-laser system activates the fluorescent beads to identify the specific cytokine and excites the PE conjugate to determine the magnitude of fluorescence, which is in direct proportion to the quantity of bound cytokine. Sensitivities range from 0.15 to 9.06 pg/ml and intra and inter assay %CV is <10%. Duplicate samples were analyzed for each animal and did not vary by more than 4%.

### qRT-PCR

Following decapitation brains were quickly dissected and hypothalamus, hippocampus and frontal cortex tissues put into RNAlater solution (Ambion, Life Technologies, CA, USA). Tissues were stored at 4 °C overnight and then transferred to −20 °C until further

processed. RNA was extracted using *mir*Vana miRNA isolation kit (Thermofisher Scientific, USA). The quality of the extracted RNA was analysed using a NanoDrop Spectrophotometer ND-1000 (Thermofisher Scientific, USA) and DNA contaminants removed using an Invitrogen TURBO DNA-*free* kit. cDNA was created using the Applied Biosystems High Capacity cDNA Reverse Transcription kit (Thermofisher Scientific, USA).

Primers were as used previously^14,18,24^. Primer sequences are listed in Supplementary Table 1. PowerUP SYBR Green Master Mix (Applied Biosystems, Life Technologies, USA) containing ROXTM Passive Reference Dye was mixed with cDNA and the appropriate primers. The qPCR reaction was carried out in fast mode (uracil-DNA polymerase activation 50 °C, 2 min; Dual-Lock DNA polymerase 95 °C, 2 s; denaturation 95 °C, 1 s; annealing/extension 60 °C, 30 s; number of cycles: 40) using QuanStudio3TM (Applied Biosystems). The transcripts were normalized to the housekeeping gene glyceraldehyde-3-phosphate dehydrogenase (GAPDH) and quantified using the ΔΔCt method, with related fold change expressed as 2^(−ΔΔCt)^. Each sample was run as a triplicate.

### Microbiome sequencing

Stool samples were collected in sterile tubes and stored at −80 C until further analysis. DNA from stool samples was extracted using the PowerSoil HTP DNA Isolation Kit (MoBio, USA) according to the manufacturer’s instructions with a beadbeater (BioSpec, USA) set on high for 2 min. The V4 region of the bacterial 16 S rRNA gene was amplified by PCR using the 515 F (AATGATACGGCGACCACCGAGATCTACACGCT) barcoded and 806 R (TATGGTAATTGTGTGYCAGCMGCCGCGGTAA) primers. For each PCR tube the following materials were added: 2 μL 515 F (forward, 10 μM) primer, 2 μL 806 R (reverse, 10 μM) primer, 25 μL PrimeSTAR Max PCR mix (Takara Bio, Shiga Prefecture, Japan), 17 μL ultra-pure water, and 4 μL of sample DNA. PCR reactions were carried out by 30 cycles of denaturation (95 °C), annealing (55 °C), and extension (72 °C), with final elongation at 72 °C. PCR products were purified using AMPure magnetic beads (Beckman Coulter, CA, USA) and quantified using a Quant-iT PicoGreen dsDNA quantitation kit (Invitrogen). Samples were pooled to 50 ng/mL, loaded on 2% agarose E-Gel (Invitrogen), purified, and sequenced using the Illumina MiSeq platform (Genomic Center, Azrieli Faculty of Medicine, Bar Ilan University, Israel).

Data analysis was performed using QIIME2^28^. Sequence reads were demultiplexed by per-sample barcodes and Illumina-sequenced amplicon read errors corrected using DADA2^29^. All analyses for mouse fecal samples were calculated based on a feature table and rarefied at 9,880 sequences. Richness was calculated using Faith’s Phylogenetic Diversity^30,31^. Beta diversity (between-sample diversity) was analyzed using unweighted UniFrac distances^32^.

### Statistical analysis

Unless otherwise specified data was analysed using one-way ANOVA with Bonferroni-corrected post hoc. A *p* value lower than 0.05 was considered statistically significant. All tests were two-tailed. Effect size is expressed as partial eta squared (n^2^p) for main effects and Hedge’s G (g) for post hoc analyses. Mean difference (MD) and standard error of differences (SED) are also provided. Results are illustrated as mean ± standard deviation (SD) unless otherwise stated. Following approaches to enhance the ability to detect sex effects in exploratory, preclinical, experiments^33^, we chose to use *a priori* non-directional groupwise contrasts disaggregated by sex. Differences between unweighted UniFrac distances were analyzed using a Pairwise Permanova test. Differences in alpha diversity were analyzed by Kruskal–Wallis (pairwise) test.

## Results

### Post-natal penicillin V induces long-term changes in social behaviour of male mice

**Figure 2:**
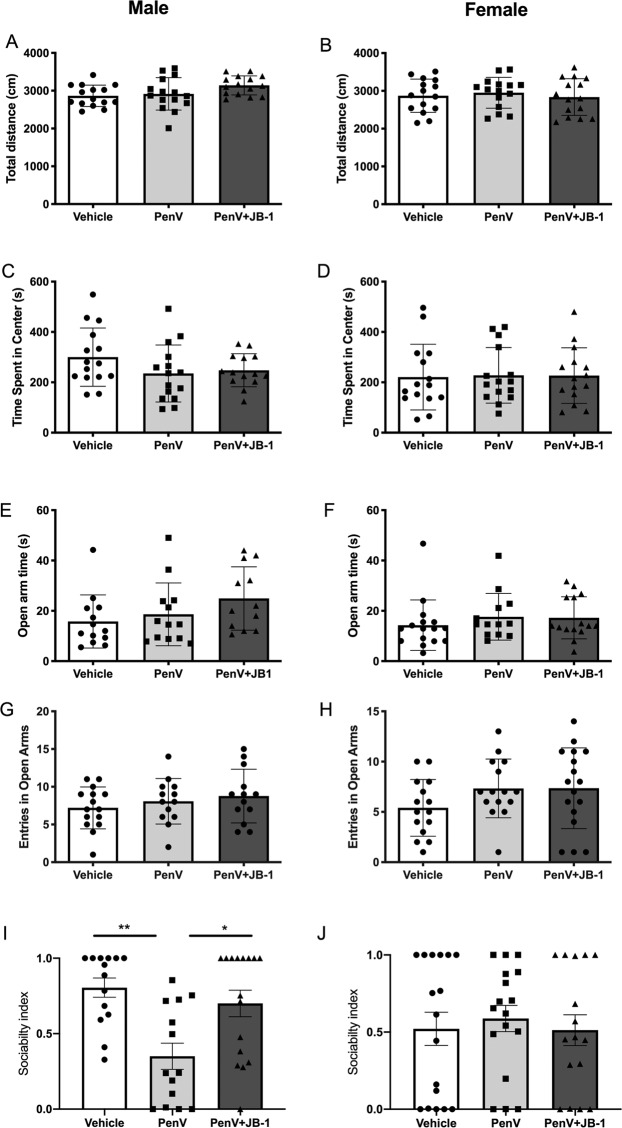
Effect of post-natal penicillin treatment on behavior. Male and female mice were treated with penicillin (PenV), penicillin and concurrent *L. rhamnosus* JB-1 (PenV + JB-1) or vehicle and tested for locomotor activity (**A**,**B**) and anxiety-like behavior in the open field (**C**,**D**) and elevated plus maze (**E**–**H**). Social behavior was assessed in the 3 chamber test (**I**,**J**). Data presented as mean ± standard deviation. (Males, n = 15 vehicle, 15 PenV, 14 PenV+JB1: Females n = 15 vehicle, 15 PenV, 15 PenV+JB-1: *p < 0.05, **p < 0.01).

Postnatal penicillin exposure had no statistically significant effect on total mobility as assessed by total distance in the open field (Fig. 2A,B) [Males, F (2,41) = 2.96, p = 0.262; Females, F (2, 42) = 0.2583, p = 0.772] nor did antibiotic treatment influence time spent in the center of the open field in either male or female mice (Fig. 2C,D) [Males, F (2, 41) = 1.722p = 0.191; Females, F (2, 42) = 0.0155, p = 0.985]. Similarly, there were no marked differences in anxiety-like behaviour in either sex as assessed in the EPM with similar numbers of entries and time spent in the open arm (Fig. 2E–H) [Male entries, F (2,41) = 0.8916, p = 0.418; Male time, F (2, 41) = 1.913, p = 0.163; Female entries, F (2,42) = 1.737, p = 0.188; Female time, F (2,42) = 0.5919, p = 0.5579]. In the assessment of social interaction, as expected, control male mice showed a clear preference for the social chamber (mean sociability index 0.80 ± 0.06). Postnatal treatment with penicillin influenced social behavior [F (2,41) =6.49, p = 0.0036, n^2^p 0.24] with post-hoc analysis indicating a significant decrease in social preference of antibiotic treated male mice compared to controls (MD = 0.45, SED = 0.12, p = 0.0011, g = 1.702) (Fig. 2I). Overall, female control mice failed to show the social preference observed in the males (mean sociability index 0.57 ± 0.1) and there were no significant differences observed in social interactions between treatment groups (Fig. 2J) [F (2, 42) = 0.179, p = 0.837].

In male & female mice, concurrent post-natal treatment with penicillin and JB-1 did not significantly alter anxiety-like behaviour as assessed in the OFT and EPM (Fig. 2A–H). However, the male mice that received both penicillin and JB-1 had a significantly higher sociability index than those treated with the antibiotic alone (0.70 ± 0.09 vs 0.35 ± 0.08, MD = 0.35, SED = 0.11, p = 0.011, g = 1.07) and did not differ significantly from control animals (Fig. 2I), indicating the JB-1 treatment counteracts the observed reduction in social behaviour cause by postnatal penicillin in male mice.

### Penicillin V treatment leads to sex dependent changes in gene expression in the brain

**Figure 3:**
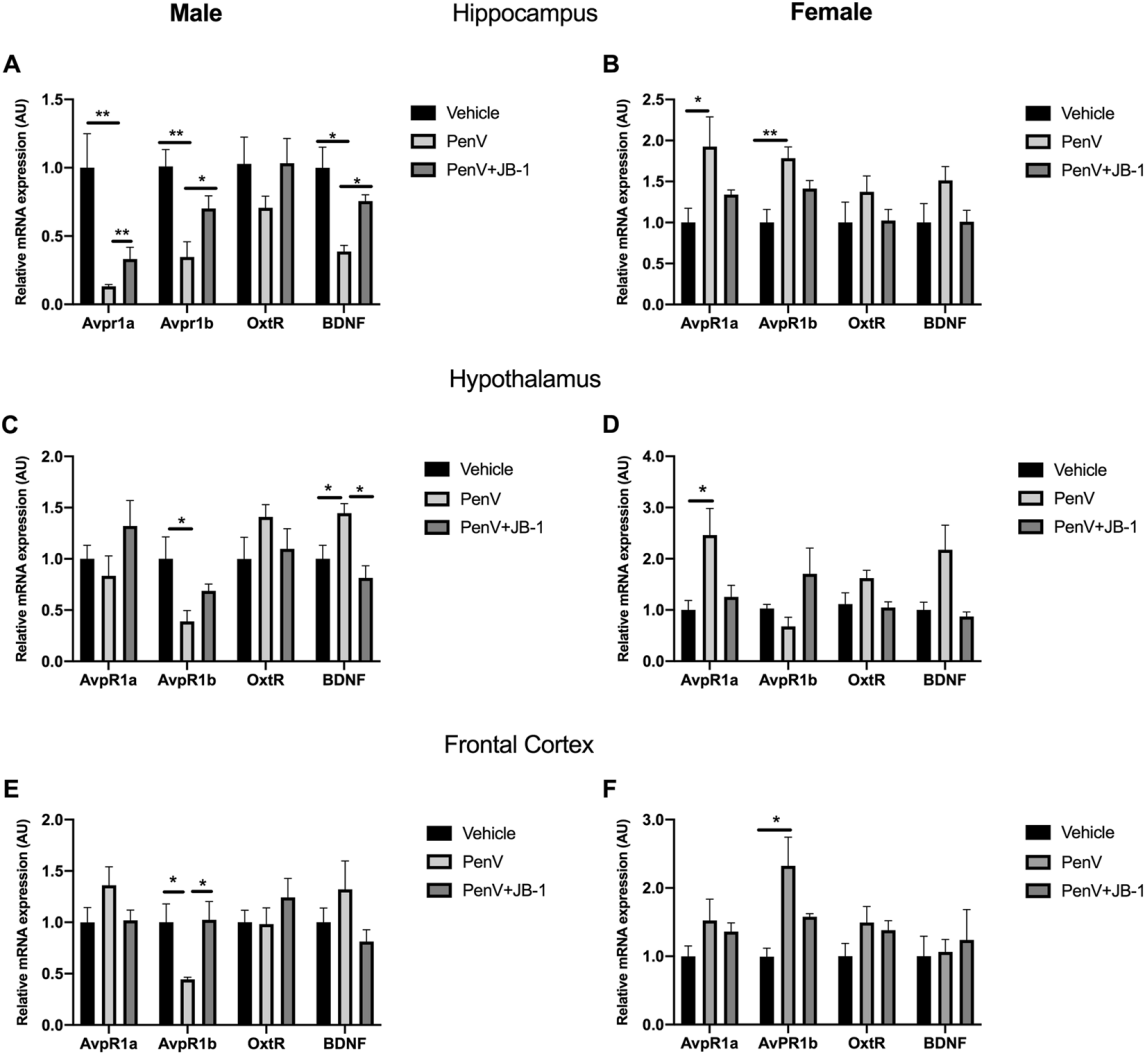
Effect of post-natal penicillin treatment on gene expression in the brain. Male and female mice were treated with penicillin (PenV), penicillin and concurrent *L. rhamnosus* JB-1 (PenV + JB-1) or vehicle and expression of Arginine Vasopressin Receptors 1a (AVPR1a) and 1b (AVPR1b), oxytocin receptor (OxtR) and brain derived neurotrophic factor (BDNF) was assessed in the hippocampus (**A**,**B**), hypothalamus (**C**,**D**) and frontal cortex (E&F). (n = 9, *p < 0.05, **p < 0.01).

Given the observed changes in social behaviour in male mice, we assessed gene expression of arginine vasopressin (AVPR) and oxytocin receptors, together with BDNF, in the hypothalamus, hippocampus and frontal cortex of mice in each treatment group. Oxytocin receptor expression did not differ significantly between treatment groups in any of the brain regions examined (Fig. 3) [Males: Hippocampus, F (2,21) = 1.337, p = 0.287, Hypothalamus, F (2, 21) = 1.40, p = 0.272, Frontal cortex, F (2, 21) = 1.134, p = 0.348; Females: Hippocampus, F (2, 21) = 1.249, p = 0.3215, Hypothalamus, F (2, 20) = 2.640, p = 0.0960, Frontal cortex, F (2, 20) = 1.845, p = 0.200]. In the hippocampus, antibiotic treatment resulted in a decrease in gene expression of AVPR 1a [F (2, 21) = 8.913, p = 0.0020, n^2^p = 0.45 post-hoc Vehicle vs PenV, MD = 0.87, SED = 0.26, p = 0.0022, *g* = 1.89] AVPR 1b [F (2, 21) = 7.809, p = 0.0043, n^2^p = 0.43, post-hoc Vehicle vs PenV, MD = 0.66, SED = 0.16,p = 0.0042, g = 2.16] and BDNF [(F (2, 21) =8.112, p = 0.0037, n^2^p = 0.43, post-hoc Vehicle vs PenV, MD = 0.61, SED = 0.15, p = 0.0026, g = 2.10] in male mice (Fig. 3A). In contrast, expression of hippocampal AVPR 1a and AVPR 1b was increased with penicillin treatment in females [AVPR 1a: F (2, 20) = 3.977, p = 0.0411, n^2^p = 0.28, post-hoc Vehicle vs PenV, MD = −0.9, SED = 0.37, p = 0.0347 g = 1.33; AVPR 1b: F (2, 21) = 13.49 P = 0.0003, n^2^p = 0.56, post-hoc Vehicle vs PenV, MD = −0.78, SED = 0.19, p = 0.0002, g = 2.29], (Fig. 3B). In the hypothalamus, penicillin treatment resulted in decreased expression of AVPR 1b [F (2,21) = 4.608, P = 0.0263, n^2^p = 0.30, post-hoc Vehicle vs PenV, MD = 0.61, SED = 0.23, p = 0.0204, g = 1.42] and increased expression of BDNF [F (2, 21) = 7.875, p = 0.0035, n^2^p = 0.42, post hoc Vehicle vs PenV,MD = −0.45, SED = 0.16, p = 0.0353, g = 1.46] in male mice (Fig. 3C) whereas in female mice only AVPR 1a expression was altered [F (2, 21) = 4.939, p = 0.0238, n^2^p = 0.31] and, in contrast to males, was again significantly increased (Vehicle vs PenV, MD = −1.46, SED = 0.49, p = 0.026, g = 1.52) (Fig. 3D). In the frontal cortex changes in gene expression were limited to AVPR 1b [Males: F (2, 21) = 5.051, p = 0.0181, n^2^p = 0.32; Females: F (2,21) = 8.080, p = 0.0034, n^2^p 0.43] and, as observed in the hippocampus, levels were decreased in males (Vehicle vs Pen V, MD = 0.56, SED 0.21, p = 0.038, g = 1.67) and increased in females (Vehicle vs Pen V, MD = −1.33, SED = 0.34, p = 0.0025, g = 1.70) (Fig. 3E,F). Overall, antibiotic treatment led to brain region and sex dependent changes in gene expression for receptors involved in modulating social behavior. In mice that were concurrently treated with penicillin and *L.rhamnosus* JB-1 there were no significant changes in gene expression when compared to control animals, suggesting that probiotic treatment prevented the effects of antibiotic on the brain in both male and female mice (Fig. 3A–F).

### Penicillin V treatment modulates tight junction protein gene expression in the brain

**Figure 4:**
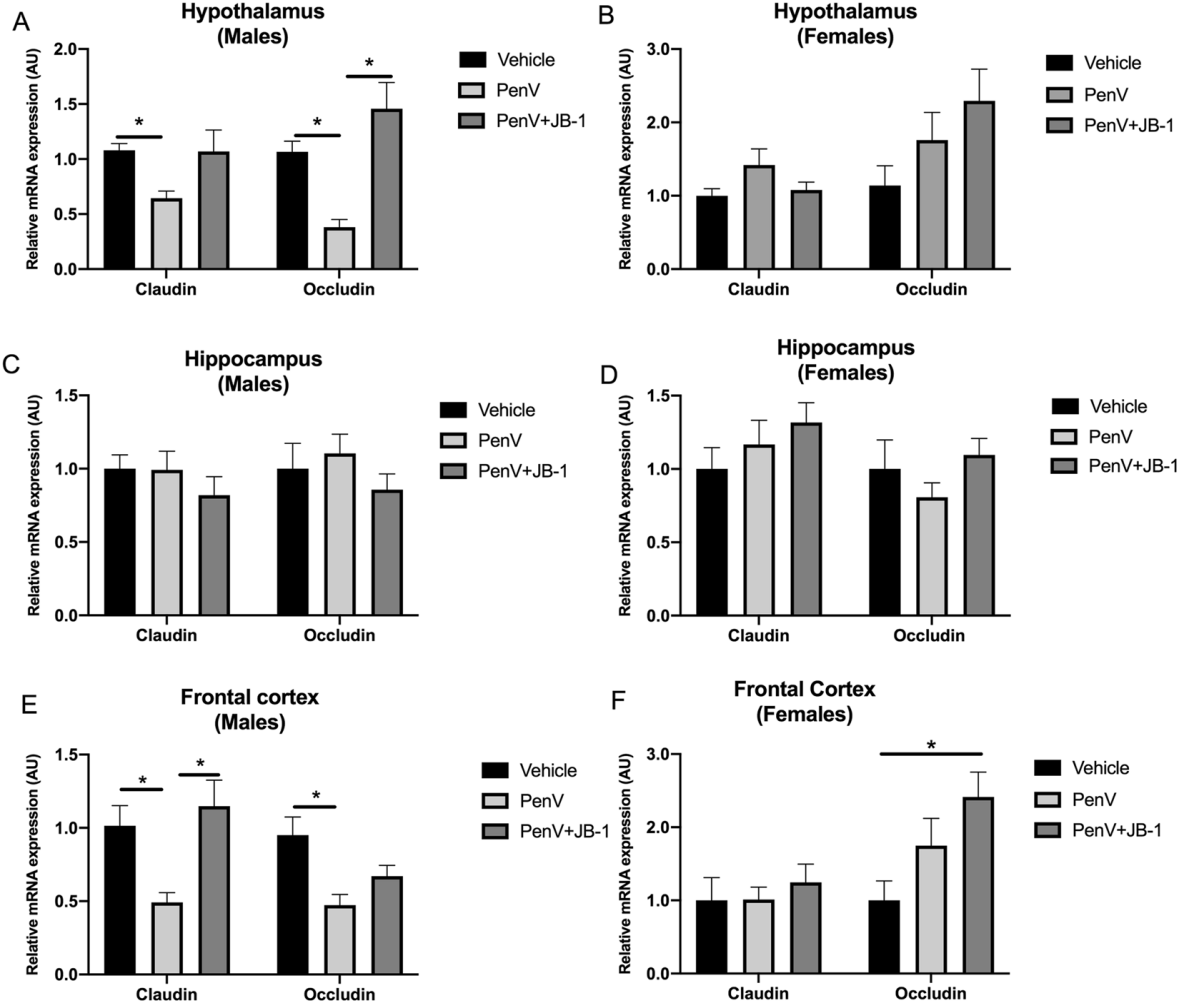
Effect of post-natal penicillin treatment brain expression of Claudin V and Occludin. Male and female mice were treated with penicillin (PenV), penicillin and concurrent *L.rhamnosus* JB-1 (PenV + JB-1) or vehicle and expression of the tight junction proteins Claudin V (Claudin) and Occludin assessed in the hypothalamus (**A**,**B**), hippocampus (**C**,**D**) and frontal cortex (**E**,**F**). (n = 9, *p < 0.05, **p < 0.01).

To assess potential changes in blood-brain-barrier we determined RNA expression of the tight junction proteins occludin, and claudin-5 (Cldn5) which are key markers of the blood brain barrier (BBB) integrity modulated by gut microbiota^34^. Here we demonstrated that male mice treated with penicillin alone had significantly reduced expression of both occludin and claudin-5 in the hypothalamus [Claudin V: F (2, 21) = 4.414, p = 0.0276, n^2^p = 0.29, post hoc, vehicle vs PenV, MD = 0.44, SED = 0.17, p = 0.045, g = 2.18; Occludin: F (2, 21) = 12.47 p = 0.0006, n^2^p = 0.54, post hoc, vehicle vs PenV, MD = 0.69, SED = 0.21, p = 0.017, g = 3.06] and frontal cortex [Claudin V: F (2, 21) = 6.597 p = 0.0071, n^2^p = 0.38, post hoc, vehicle vs PenV, MD = 0.45, SED = 0.18, p = 0.034, g = 1.80; Occludin: F (2, 21) = 6.579, p = 0.0060, n^2^p = 0.38, post hoc, vehicle vs PenV, MD = 0.48, SED = 0.13, p = 0.0045, g = 1.85] compared to control animals (Fig. 4A,E). These reductions in gene expression were not observed in females (Fig. 4B,F). No significant differences in tight junction gene expression were observed in the hippocampus between treatment groups of either male [Claudin V: F (2, 21) = 0.8164, p = 0.4596; Occludin: F (2, 21) = 0.7580, p = 0.4838] or female [Claudin V: F (2, 21) = 1.172 p = 0.3404; Occludin: F (2, 21) = 1.103, p = 0.3611] mice (Fig. 4C,D). Concurrent treatment with penicillin and JB-1 did not result in any difference in gene expression compared to control animals with the exception of occludin expression in the frontal cortex of females, [F (2, 21) = 4.598, p = 0.0329, n^2^p = 0.30, post hoc vehicle vs PenV+JB-1, MD = −1.4, SED = 0.46, p = 0.026, g = 2.06] which was significantly increased compared to controls (Fig. 4B).

### Penicillin V treatment results in sex dependent changes in immune cell populations

**Figure 5:**
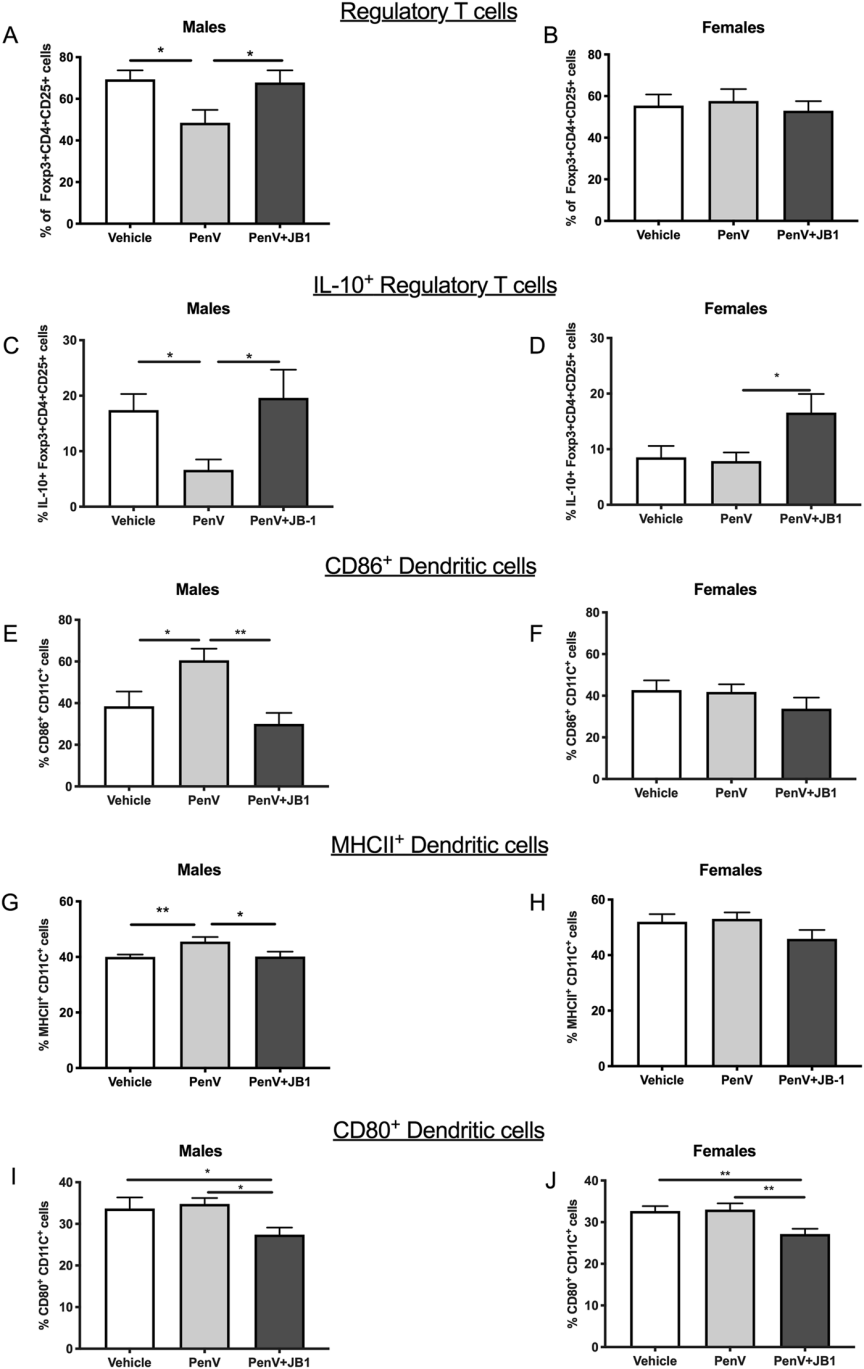
Effect of post-natal penicillin treatment on immune cell populations. Male and female mice were treated with penicillin (PenV), penicillin and concurrent *L.rhamnosus* JB-1 (PenV + JB-1) or vehicle and flowcytometry used to determine proportion of CD4^+^CD25^+^ splenocytes expressing Foxp3 (**A**,**B**), and CD4 + CD25 + Foxp3+ cells expressing IL-10 (**C**,**D**). The proportion of dendritic cells (CD11c+) expressing CD86 (**E**,**F**), MHCII (**G**,**H**) and CD80 (**I**,**J**) were also assessed (n = 12, *p < 0.05, **p < 0.01).

Given the potential link between the peripheral immune environment, particularly the inflammatory/anti-inflammatory balance, and brain function and behavior, we also assessed several immune parameters in our treatment groups. T regulatory cells play a major role in immunomodulation, are generally anti-inflammatory and have been associated with modulating behavior^35,36^. We observed, as with brain and behavior effects, there were sex dependent changes in the population of T regulatory cells following penicillin treatment. The proportion of CD4 + CD25 + cells that also express the Foxp3, a marker of regulatory T cells, was significantly decreased in the AB group compared to controls in males [F (2, 41) = 4.442 p = 0.0178, n^2^p = 0.18, post hoc Vehicle vs PenV, MD = 20.91, SED = 7.82, p = 0.017, g = 1.00] but not females [F (2, 42) = 0.1817, p = 0.8344] (Fig. 5A,B). Furthermore, within the CD4 + CD25 + Foxp3+ cell population there was a reduced proportion of cells expressing the immunoregulatory cytokine IL-10, in male [F (2, 40) = 4.262, p = 0.0210, n^2^p = 0.17, post hoc Vehicle vs PenV, MD = 6.56, SED = 3.15, p = 0.02, g = 1.14] but not female [F (2, 42) = 4.014, p = 0.0234, n^2^p = 0.16, post hoc Vehicle vs PenV, p = 0.842] penicillin treated mice (Fig. 5C,D).

We also assessed expression of activation markers on dendritic cells, antigen presenting cells that play an important role in shaping immune responses. Here we observed that male mice treated with penicillin alone had a higher proportion of CD11c + cells expressing MHCII [F (2, 40) = 4.703, p = 0.0146, n^2^p = 0.19, post hoc Vehicle vs PenV, MD = −5.5, SED = 2.0, p = 0.0099, g = 1.07] and the co-stimulatory molecule, CD86 [F (2, 40) = 6.630, p = 0.0033, n^2^p = 0.24, post hoc Vehicle vs PenV, MD = −22.0, SED = 8.4, p = 0.0126, g = 0.89] both indicators of DC activation (Fig. 5E,F). These changes were not evident in female penicillin treated mice [MHCII: F (2, 42) = 1.899 p = 0.1596; CD86: F (2, 42) = 1.116, p = 0.3351] (Fig. 5G,H). As with behaviour and gene expression in the brain, changes occurring following antibiotic treatment were not observed in the group treated with both JB-1 and penicillin. However, treatment with antibiotic and JB-1 did lead to a significant decrease in expression of another costimulatory molecule and marker of DC activation, CD80, in both male [F (2, 40) = 3.598 p = 0.0366, n^2^p = 0.15, post hoc Vehicle vs PenV+JB1, MD = 6.2, SED = 2.9, p = 0.0389, g = 0.7 PenV vs PenV+JB1, MD = 7.4, SED = 2.9, p = 0.0160, g = 1.2] and female [F (2, 42) = 5.854, p = 0.0050, n^2^p = 0.22, Vehicle vs PenV+JB1,MD = 5.5, SED = 1.9, p = 0.0053, g = 1.00 PenV vs PenV + JB1, MD = 5.9, SED = 1.9, p = 0.0032, g = 1.1] mice compared to the other treatment groups (Fig. 5I,J) and a significant increase in the CD4 + CD25 + Foxp3 + IL10 + population in females compared to the control group (MD = −8.0, SED = 3.4, p = 0.022 g = 0.67) and those treated with penicillin alone (MD = −8.7, SED = 3.4, p = 0.014, g = 0.50) (Fig. 5D).

Serum cytokines did not differ significantly between treatment groups in either male or female mice (Supplementary Table 2)

### Postnatal treatment with penicillin V leads to disruption of the gut microbiome

**Figure 6:**
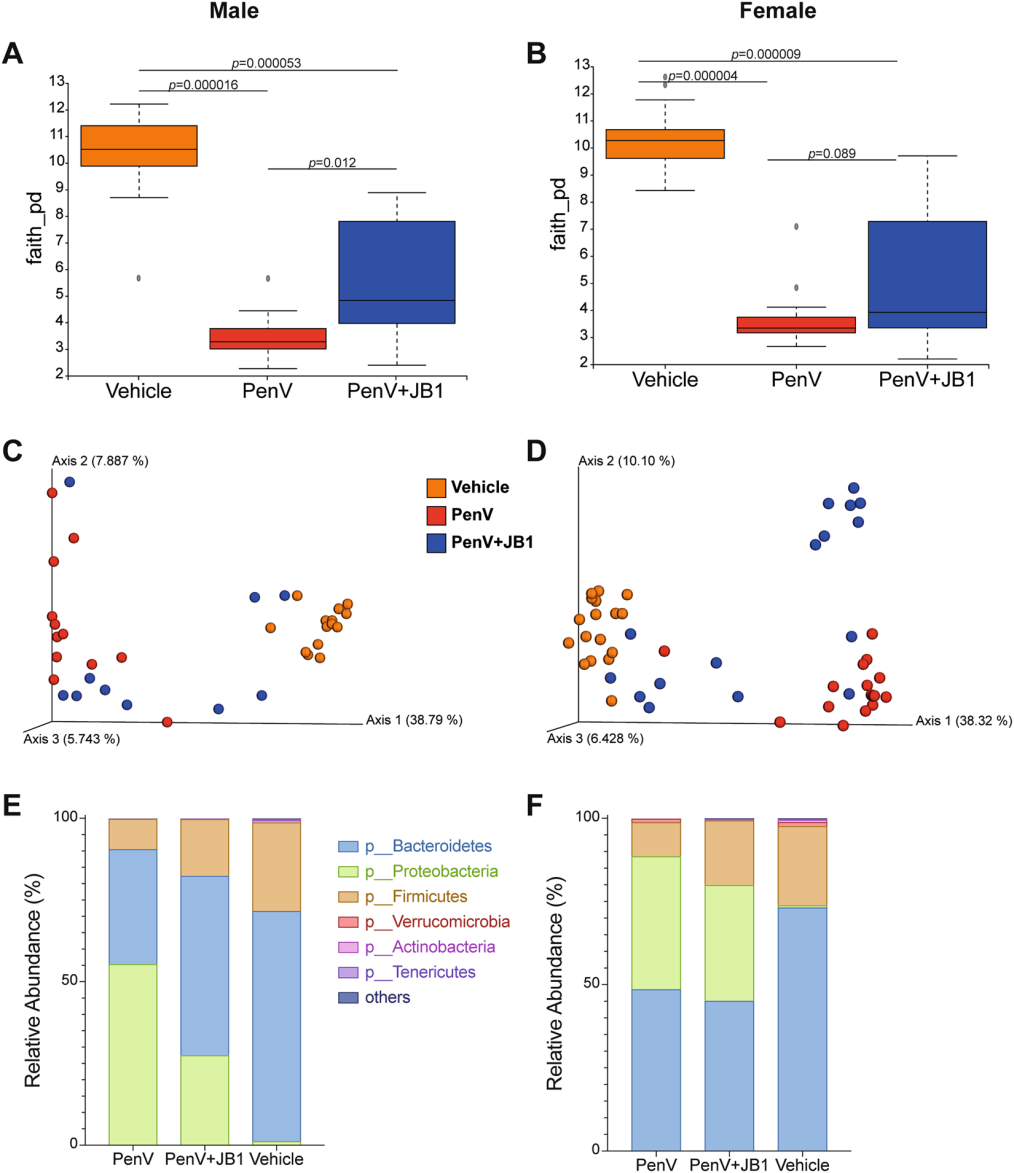
Fecal Microbiome composition at 3 weeks. Alpha diversity calculated using Faith’s PD (**A**,**B**), beta-Diversity calculated using unweighted UniFrac matrix (**C**,**D**) and relative abundance of bacteria phyla, expressed in percentage (**E**,**F**) in male and female mice immediately following treatment with penicillin, penicillin and concurrent *L.rhamnosus* JB-1 (PenV + JB-1) or vehicle from postnatal day 14–21 (Males, n = 11 vehicle, 12 PenV, 12 PenV + JB1: Females n = 15 vehicle, 15 PenV, 15 PenV + JB-1).

**Figure 7:**
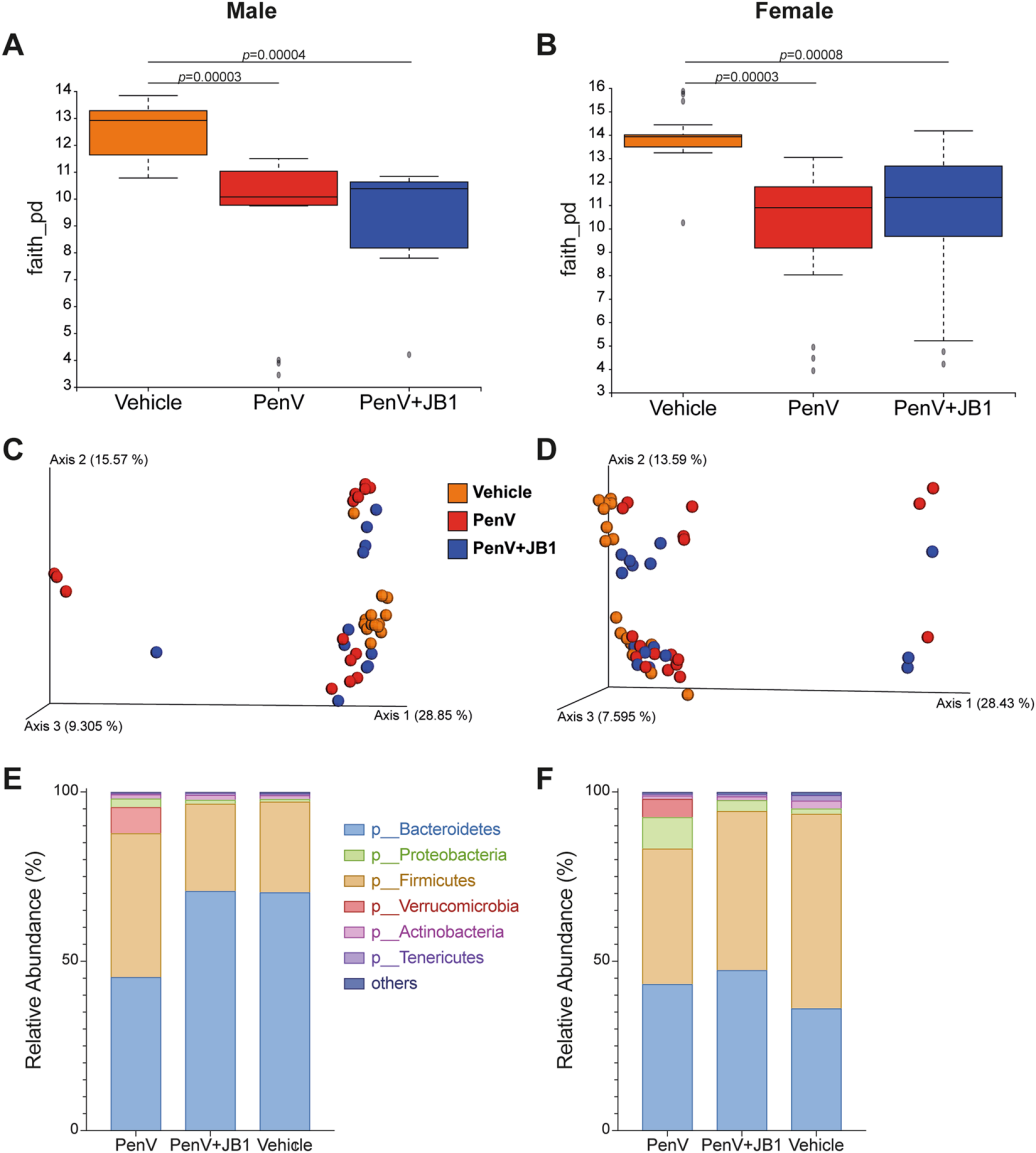
Fecal Microbiome composition at 6 weeks. Alpha diversity calculated using Faith’s PD (**A**,**B**), beta-Diversity calculated using unweighted UniFrac matrix (**C**,**D**) and relative abundance of bacteria phyla, expressed in percentage (**E**,**F**) in male and female mice 3 weeks following treatment with penicillin (PenV), penicillin and concurrent *L.r hamnosus* JB-1 (PenV + JB-1) or vehicle from postnatal day 14–21 (Males, n = 15 vehicle, 14 PenV, 12 PenV + JB1: Females n = 15 vehicle, 15 PenV, 15 PenV + JB-1).

The microbiota of the mice was assessed in fecal samples collected on the final day of antibiotic treatment (3 weeks old) and 3 weeks later at initiation of behavioral testing (6 weeks old). Directly following treatment the microbiome of mice receiving either penicillin alone or penicillin with JB-1 was significantly less diverse (alpha-diversity) than the control group (Fig. 6A,B) and also clustered separately from the controls (beta-diversity, unweighted UniFrac) in both male (PenV vs vehicle, p = 0.011; PenV/JB-1 vs vehicle, p = 0.011) and female (PenV vs vehicle, p = 0.001; PenV/JB-1 vs vehicle, p = 0.001) mice (Fig. 6C,D). While lower than controls, the alpha-diversity of mice treated concurrently with JB-1 and penicillin had significantly higher diversity than those treated with penicillin alone (Fig. 6A,B). At 3 weeks following cessation of treatment, diversity increased in both groups receiving penicillin but was still significantly less diverse (Fig. 7B), and clustered separately from control mice in both males (PenV vs vehicle, p = 0.001; PenV/JB-1 vs vehicle, p = 0.001) and females (PenV vs vehicle, p = 0.001; PenV/JB-1 vs vehicle, p = 0.002) (Fig. 7C,D) indicating a degree of dysbiosis remained 3 weeks after cessation penicillin treatment.

Assessment of relative abundance at the phyla level directly following treatment revealed mice exposed to penicillin were characterized by a large increase in the abundance of Proteobacteria compared to vehicle controls, whether receiving antibiotic alone (males, p < 0.0001, g = 2.48; females p < 0.0001, g = 4.50) or concurrently with JB-1 (males, p = 0.002, g = 1.21; females, p < 0.0001, g = 1.53) (Fig. 6E,F). There was also a corresponding decrease in abundance of Bacteroidetes in both treatment groups in females (PenV vs vehicle, p = 0.0002, g = 1.34; PenV/JB-1 vs vehicle, p = 0.0008, g = 1.23) but this only reached significant in males when penicillin alone was compared to control (p = 0.0023, g = 1.16). At 3 weeks following antibiotic treatment there were no longer significant differences in abundance of Proteobacteria or Bacteroidetes between groups (Fig. 7E,F). However, male mice treated with antibiotic alone had a significantly greater abundance of Firmicutes compared to control mice (p = 0.01, g = 0.96) or those receiving concurrent antibiotic and probiotic (p = 0.0148, g = 0.89).

## Discussion

Antibiotics are commonly, sometimes unnecessarily, prescribed in pediatric care^37,38^. Studies have suggested that early life dysbiosis associated with antibiotic use has potential long-term consequences on health^39^, including poorer neurocognitive outcomes^40^. However, little is known regarding impact of antibiotic use on the gut-brain axis and potential implications for mental health. In this study, we demonstrate that a clinically relevant dose of penicillin, given for one week immediately prior to weaning, causes long-term changes in brain, behaviour and peripheral immune cell populations in mice. These effects were sex-specific, being most marked in males and were attenuated with concurrent supplementation with *L. rhamnosus* JB-1.

The current study focused on direct treatment of pups with penicillin V administered from post-natal day 14 to 21. Directly following the treatment period alpha diversity was significantly decreased in male and female antibiotic exposed groups, regardless of concurrent probiotic treatment. Changes in abundance at the phyla level were similar between sexes and most marked by an increase in Proteobacteria and decrease in Bacteroidetes. Three weeks following treatment there were no longer significant differences in abundance of Proteobacteria and decrease in Bacteroidetes. However, while diversity increased in mice that received penicillin alone or penicillin and JB-1, they did not return to control levels indicating there was still some degree of dysbiosis in both male and female mice at the time of behavioural testing.

In assessing behaviour, we found a decrease in sociability in male mice exposed to penicillin, but no significant difference in anxiety-like traits in either male or female mice. Previous studies have demonstrated that maternal penicillin exposure, throughout pregnancy, or through pregnancy and weaning, leads to a reduction in both sociability and anxiety-like behavior^18,41^. This suggests there may be distinct pathways modulating sociability and anxiety-like behaviours in response to penicillin, with changes in anxiety dependent on intrauterine exposure or factors associated with maternal dysbiosis.

The changes in social behaviour of male mice following treatment with penicillin was associated with alterations in brain gene expression; principally decreased expression of AVPR1b in the hypothalamus, hippocampus and frontal cortex. Hippocampal AVPR1a was also decreased. It seems likely that changes in AVPR are causally related to the effects of penicillin treatment on behavior as both AVPR1a and 1b play important roles in promoting social interaction and social motivation^42–46^.

Of note, AVPR1a and 1b expression was also changed with antibiotic treatment in female mice, but expression was increased rather than decreased. This may reflect sex differences in vasopressinergic pathways. For example, AVPR1a has a greater role in modulating anxiety in male mice^47,48^ and blocking this receptor alters social play in males and females in an opposite fashion^49^. Similarly, sex related differences in AVPR1b dependent brain responses have been reported^50^. Altered AVPR expression is a consistent finding related to early-life penicillin exposure^18,41^ and it is possible that sex differences in the vasopressinergic system may contribute to the sex dependent nature of the effects of this antibiotic on behaviour. However, caution must be applied in interpreting the effects on social behaviour in females as, in keeping with previous reports of reduced social interactions in females compared to males^41,51^, there was no social preference observed in female control animals.

The BBB plays an important role in maintaining homeostasis of the CNS and protecting against exposure to pathogens and potential toxins coming from the blood. The barrier capability is dependent on tight junction proteins, the function of which can be influenced by gut microbes^34^. Penicillin treatment led to a significant decrease in expression of two major tight junction proteins, claudin 5 and occludin, in the hypothalamus and frontal cortex of males, but not females. This is suggestive of reduced barrier function in male mice consequent to early-life antibiotic exposure. Decreased expression of tight junction proteins in the CNS has previously been associated with stress vulnerability and correlated with social avoidance behaviour in response to chronic social defeat stress in mice^52^. A decrease in expression of tight junction markers has also been described in germ-free mice^34^. In contrast to our current findings, exposure to penicillin through maternal treatment was demonstrated to enhance tight junction expression in the hippocampus of offspring^18^. This disparity is likely related to the stage of BBB development at which the intervention occurs. Antibiotic exposure during the intrauterine period corresponds with the initial development of the BBB, which begins around embryonic days E11 to E13^53^, and it is perhaps not surprising that microbiome disruption during this very early point influences tight junction expression differently than when the BBB is more established. Our current study cannot determine the mechanism underlying the effect of penicillin on expression of tight junction proteins. Inflammation can increase BBB permeability^54^. However, while we observed changes in immune cells populations that suggest a more pro-inflammatory environment in antibiotic treated males, circulating cytokines did not indicate overt systemic inflammation in these animals 4 weeks following antibiotic treatment. Nevertheless, it should be noted that we cannot rule out increased inflammation at an earlier time point influencing BBB development.

There is a clear relationship between the microbiota-gut-brain axis and the immune system^55^, with changes in immune cell populations being associated with modulation of gut-brain signaling^26^. In keeping with these previous findings, the current study identified long-term systemic immune changes, at the cell population level, associated with behavioral and brain gene expression changes in penicillin treated male mice. Four weeks following cessation of treatment, male mice receiving antibiotic alone exhibited a greater proportion of activated DCs, as determined by expression of CD86 and MHCII, suggesting sustained changes in innate immunity. Penicillin treated male mice also exhibited a decrease in the proportion of the CD4 + CD25 + cells expressing Foxp3 + , a characteristic of the regulatory T cells which play an important role in suppressing immune responses. Treatment with penicillin alone also decreased the proportion of T regulatory cells expressing the immunoregulatory cytokine IL-10. Overall, these changes suggest a proinflammatory bias of the immune system in male mice treated with penicillin alone, although circulating cytokine levels did not suggest active systemic inflammation. While we cannot determine causality between the observed immune changes and CNS effects, it is interesting to note that regulatory T cells can influence behaviour^36,56^ and mediate resistance to behavioral effects of chronic stress exposure in mice^35^. Future studies should include a detailed examination of the relationship between the loss of immunoregulatory function and the effects of microbiome disruption on the brain.

There is increasing evidence that the consequences of early-life microbiome disruption on the brain and behaviour are sex dependent. Maternal treatment with penicillin leads to distinct behavioral changes and brain gene expression patterns in male and female offspring^18,41^, while CNS associated differences in germ-free mice are sex-specific and reconstitution of a normal microbiota restores anxiety-like behaviour in males but not in females^57^. Our findings further support the concept that response to dysbiosis in early life is sex dependent, with changes in behaviour, gene expression and immune cell populations most marked in males. The factors determining the sex differences in response to disruption of the gut microbiota are unclear. While the influence of sex hormones is an obvious factor to consider there may be other differences in the immune or neuronal elements of gut-brain signaling that account for the distinct outcomes of early-life dysbiosis in males and females.

The observed changes in behaviour and gene expression are unlikely to be due to direct effects of penicillin on the brain as renal clearance of penicillin is rapid and dissemination to the cerebral spinal fluid occurs at very low levels^58^. We cannot completely rule out the possibility of direct effects of penicillin on the enteric nervous system (ENS)^59^, that can in turn influence gut-brain signaling^60^. However, the dose of penicillin used in this study is significantly lower than that which has been demonstrated to modulate ENS activity as determined by changes in gut motility^60^.

*L. rhamnosus* JB-1 has anxiolytic and antidepressant-like effects in mice^24,61^ and prevents behavioral effects of chronic stress exposure^23^. JB-1 has also been demonstrated to partially attenuate the long-term impact of maternal penicillin treatment on brain and behaviour of offspring^18^. For these reasons, and given that we have already extensively reported on the effects of JB-1 treatment alone in BALB/c mice, this study was designed to assess the effect of concurrent JB-1 treatment on postnatal penicillin exposure. The reduction in social behaviour associated with penicillin treatment did not occur in those male mice that received concurrent JB-1. Furthermore, the majority of changes in gene expression, and all effects in immune cell populations, caused by penicillin alone were prevented by JB-1 and none of these parameters differed significantly between control mice and those receiving concurrent penicillin and JB-1. These findings add to existing data suggesting that probiotic treatment can mitigate potential detrimental effects of dysbiosis caused by antibiotics^18^. We did not explore the mechanism through which JB-1 counters the effects of penicillin on the brain and immune system, and a limitation of the study is the absence of a treatment group receiving probiotic alone. However, there are several factors that could contribute to the mitigation of antibiotic effects by JB-1. While, immediately following the treatment period, the gut microbial diversity was decreased in mice treated with penicillin alone and those receiving both penicillin and JB1, the loss in diversity was significantly reduced in mice receiving concurrent antibiotic and probiotic.

It is possible that this partial attenuation of penicillin induced dysbiosis could contribute to JB-1 mediated protection against the effects of antibiotic exposure on the brain and immune system. However, it is also reasonable to speculate that direct JB-1/host interactions are responsible for the probiotic effects. In BALB/c mice, feeding of JB-1 induces a tolerogenic phenotype in DCs that in turn drive the production of functional T regulatory cells^62,63^. The loss of T regulatory cells in penicillin treated male mice may be counteracted by these immunoregulatory actions of JB-1, thus influencing any immune contributions to the effects of dysbiosis on brain and behaviour. It is possible that immunomodulatory effects of JB-1, independent of penicillin treatment, mask the impact of the antibiotic. Such independent effects are suggested by the observed decrease in CD80 expression in dendritic cells of both males and females with JB-1 treatment in the absence an effect of antibiotic treatment on this marker. Similarly, treatment with JB-1 increases the IL-10 expressing T regulatory cell population compared to controls and mice receiving penicillin alone. JB-1 in the intestine can also activate the vagus nerve through a mechanism that is unlikely to depend on the existing microbiota^60^, and vagus nerve signaling has been demonstrated to be essential to the anxiolytic and antidepressant effects of the bacteria^24^. However, until further studies are conducted, we can only speculate regarding the contributions of microbiome modulation, immunoregulation and vagal signaling to the ability of JB-1 to counteract the effects of penicillin on brain and behaviour.

In assessing the overall pattern of effects of low dose post-natal penicillin exposure (Table 1), it can be seen that, despite very similar disruption of the gut microbiome, antibiotic treatment leads to distinct changes in the brain, behavior, and immune profile of male and female mice. Penicillin treatment leads to a reduction in social behaviour only in male mice that is accompanied by altered gene expression in the brain, most markedly a decrease in AVPR 1b expression in all regions assessed, and changes in peripheral immune cells suggesting a proinflammatory bias. No such changes are observed in mice given concurrent antibiotic and probiotic treatment. This study demonstrates, for the first time, that persistent effects of clinically relevant doses of antibiotic on brain and behaviour do not require intrauterine exposure^18,41^ and dysbiosis during postnatal development can have long term implications. We add support for the need to consider the possible negative long-term effects of early-life antibiotic exposure in relation to neuropsychiatric disorders. Our data further indicates that probiotic based strategies may provide a means to counteract the potential detrimental CNS effects of antibiotics and as such should be a focus of future research.

**Table 1:** Overview of the effects of post-natal penicillin V treatment in male and female mice compared to vehicle treated controls.

			Males		Females	
			PenV	PenV+JB1	PenV	PenV+JB1
Behaviour	Anxiety-like		—	—	—	—
	Social interaction		↓	—	—	—
Brain gene expression	AVPR 1a	HPC	↓	—	↑	—
		HPT	—	—	↑	—
		FC	—	—		
	AVPR 1b	HPC	↓	—	↑	—
		HPT	↓	—	—	—
		FC	↓	—	↑	—
	OxtR	HPC	—	—	—	—
		HPT	—	—	—	—
		FC	—	—	—	—
	BDNF	HPC	↓	—	—	—
		HPT	↑	—	—	—
		FC	—	—	—	—
	Claudin-5	HPC	—	—	—	—
		HPT	↓	—	—	—
		FC	↓	—	—	—
	Occludin	HPC	—	—	—	—
		HPC	↓	—	—	—
		FC	↓	—	—	↑
Immune parameters	Foxp3 + T cells		↓	—	—	—
	IL-10 + Foxp3 + T cells		↓	—	—	↑
	CD80 + Dendritic cells		—	↓	—	↓
	CD86 + Dendritic cells		↑	—	—	—
	MHCII + Dendritic cells		↑	—	—	—
Microbiome	α-Diversity	Week 3	↓	↓	↓	↓
		Week 6	↓	↓	↓	↓
	Proteobacteria	Week 3	↑	↑	↑	↑
		Week 6	—	—	—	—
	Bacteroidetes	Week 3	↓	—	↓	↓
		Week 6	—	—	—	—

↑Increased, ↓Decreased, —Unchanged. Abbreviations; AVPR, arginine vasopressin receptor; BDNF, brain derived neurotrophic factor; FC, frontal cortex; HPC, hippocampus; HPT, hypothalamus; JB-1, *Lactobacillus rhamnosus* JB-1; OxtR, oxytocin receptor; PenV, penicillin V.

## Supplementary information

Supplementary Information.
