# Publisher Correction: Sex dependent effects of post-natal penicillin on brain, behavior and immune regulation are prevented by concurrent probiotic treatment

**DOI:** 10.1038/s41598-020-75606-4

**Published:** 2020-10-22

**Authors:** Marya Kayyal, Tanvi Javkar, M. Firoz Mian, Dana Binyamin, Omry Koren, Karen-Anne McVey Neufeld, Paul Forsythe

**Affiliations:** 1 McMaster Brain-Body Institute at St Joseph’s Healthcare Hamilton, Hamilton, Canada; 2 The Azrieli Faculty of Medicine, Bar-Ilan University, Safed, Israel; 3 Department of Pathology and Molecular Medicine, McMaster University, Hamilton, Canada; 4 Department of Medicine, McMaster University, Hamilton, Canada; 5 Firestone Institute for Respiratory Health, St Joseph’s Healthcare Hamilton, Hamilton, Canada

Correction to: *Scientific Reports* https://doi.org/10.1038/s41598-020-67271-4, published online 25 June 2020

In the original version of this Article, the author Karen-Anne McVey Neufeld was incorrectly indexed. This error has now been corrected.

