# Supplementary material for: Sex dependent effects of post-natal penicillin on brain, behavior and immune regulation are prevented by concurrent probiotic treatment
Source: Sci Rep. 2020 Jun 25;10:10318. doi: 10.1038/s41598-020-67271-4 (PMC7316860; doi:10.1038/s41598-020-67271-4)
Supplement: Supplementary file 1 — Supplementary Information. [file 41598_2020_67271_MOESM1_ESM.docx]

**Sex dependent effects of post-natal penicillin on brain, behavior and immune regulation are prevented by concurrent probiotic treatment.**

Marya Kayyal^1^, Tanvi Javkar^1^, M. Firoz Mian^1^, Dana Binyamin^2^, Omry Koren^2^, Karen-Anne McVey Neufeld^1,3^, Paul Forsythe ^1,4,5*^

1. McMaster Brain-Body Institute at St Joseph's Healthcare Hamilton, Hamilton, Ontario Canada.
2. The Azrieli Faculty of Medicine, Bar-Ilan University, Safed, Israel.
3. Department of Pathology and Molecular Medicine, McMaster University, Hamilton, Ontario, Canada.
4. Department of Medicine, McMaster University, Hamilton, Ontario, Canada.
5. Firestone Institute for Respiratory Health, St Joseph's Healthcare Hamilton, Hamilton, Ontario, Canada.

***Address for correspondence**

Paul Forsythe, Ph.D.

McMaster University

Department of Medicine

St. Joseph's Healthcare

50 Charlton Avenue East, T3302

Hamilton, Ontario L8N 4A6

E-mail: forsytp@mcmaster.ca

**Supplemental Material**

| **Target mRNA** | **Forward (F) and Reverse (R) Primers** |
| --- | --- |
| GAPDH | F: 5’-TGG CCT CCA AGG AGT AAG AAA C-3’  R: 5’-GGG ATA GGG CCT CTC TTG-3’ |
| AVPR1a | F: 5’-GGG ATA CCA ATT TCG TTT GG-3’  R: 5’-AAG CCA GTA ACG CCG TGA T-3’ |
| AVPR1b | F: 5’-TCT ACT CTC CGT CTT AGC CTT AAC CT-3’  R: 5’-CTC CAT CCA CCT GCT CCA A-3’ |
| BDNF | F: 5’-CTG ACA CTT TTG AGC ACG TCA TC-3’  R: 5’-CAC CCG GGA AGT GTA CAA GTC-3’ |
| OxtR | F: 5’‐CTG GTT CTG ATC CTG TGG T‐3′,  R: 5’‐TGT CTC AGG TCC TTC TCC T‐3′ |
| Claudin 5 | F: 5’ -TCA GCT TCC CGG TCA AGT ACT C-3’  R: 5’- CCG CCC TTA GAC ATA GTT CTT CTT-3’ |
| Occludin | F: 5’- TGA ACA GCC CCC CAA TGT -3’  R: 5’ -TCA ACT CTT TCC GCA TAG TCA GAT-3’ |

**Supplementary Table 1.** Primer sequences for qRT-PCR.Abbreviations: GAPDH: glyceraldehyde 3-phosphate dehydrogenase, AVPR: arginine vasopressin receptor, BDNF: brain-derived neurotrophic factor, OxtR: Oxytocin receptor

|  | Treatment groups | | | | | |
| --- | --- | --- | --- | --- | --- | --- |
|  | Male | | | Female | | |
| Cytokine (pg/ml) | Vehicle | PenV | PenV+JB1 | Vehicle | PenV | PenV+JB1 |
| IFNγ | 1.1 ± 0.3 | 1.2 ± 0.4 | 2.5 ±0.9 | 1.8 ±0.6 | 1.3 ±0.4 | 2.3 ±0.7 |
| IL-1β | 6.1 ±1.3 | 3.8 ±0.7 | 3.5 ±0.5 | 2.7 ±0.5 | 4.2 ±0.9 | 3.1 ±1.2 |
| IL-2 | 1.6 ±0.4 | 1.6 ±0.3 | 1.8 ±0.6 | 3.2 ±0.9 | 1.0 ±0.2 | 1.4 ±0.2 |
| IL-4 | 0.5 ±0.1 | 0.6 ±0.2 | 0.6 ±0.2 | 0.4 ±0.05 | 0.3 ±0.06 | 0.7 ±0.2 |
| IL-6 | 2.9 ±1.9 | 2.6 ±0.7 | 1.6 ±0.6 | 2.9 ±0.6 | 4.8 ±2.6 | 1.6 ±0.4 |
| IL-10 | 6.5 ±1.7 | 5.3 ±1.7 | 6.5 ±2.5 | 4.4 ±1.2 | 13.2 ±8.3 | 5.9 ±1.7 |
| IL-12 | 32.7 ±13.2 | 32.9 ±13.0 | 17.1 ±9.0 | 10.5 ±4.0 | 13.2 ±5.4 | 13.5 ±2.1 |
| MCP-1 | 8.4 ±1.4 | 14.7 ±2.6 | 12.2 ±2.2 | 12.2 ±2.3 | 6.3 ±2.2 | 19.4 ±2.7 |
| TNF | 7.5 ±1.0 | 6.8 ±0.7 | 6.7 ±0.8 | 7.5 ±0.4 | 5.4 ±0.6 | 7.7 ±0.7 |

**Supplementary Table 2.** Serum cytokine levels in male and female mice treated with penicillin (PenV), penicillin and concurrent *L.rhamnosus* JB-1 (PenV+JB-1) or vehicle , presented as mean ± standard error of the mean (n=8). Abbreviations: IL: interleukin, TNF: tumor necrosis factor, IFN-γ: interferon-gamma, MCP-1: Monocyte chemoattractant protein 1 (CCL2),

**A**

**B**

**Supplementary Figure 1.** Gating strategies for flow cytometric analysis of T regulatory cells (A) and dendritic cells (B).
